# Case Report: A case of Kounis syndrome induced by iodine contrast agent during coronary angiography

**DOI:** 10.3389/fcvm.2024.1355692

**Published:** 2024-03-05

**Authors:** Yangliu Sun, Jian Zhang

**Affiliations:** Department of Cardiovascular Medicine, The First Hospital of Jilin University, Changchun, China

**Keywords:** allergy, coronary spasm, ioversol, Kounis syndrome, cardiovascular system

## Abstract

Kounis Syndrome (KS), a seldom-seen adverse reaction to iodine contrast agents, has an incidence that remains unclear. At present, there are no unified guidelines for managing KS either nationally or internationally. Ioversol, a new triiodinated hypotonic nonionic contrast agent, is commonly used in cardiovascular Computed Tomography (CT) and vascular imaging for diagnostic purposes. Its principal adverse reactions encompass fever, dermatological responses, convulsions, respiratory distress, hypersensitivity reactions including KS, and acute renal injury. This paper documents a case of KS induced by an iodine contrast agent during coronary angiography and, at the same time, searches for related literature and carries out a summary analysis in an attempt to provide a dependable reference for clinicians to make accurate diagnoses and treatments.

## Introduction

As an acute coronary condition, Kounis Syndrome (KS) results from an anaphylactic reaction caused by the interaction of mast cells and inflammatory cells. It extends beyond a single-organ disorder, representing a complex, multisystem disease with considerable implications for both morbidity and mortality (1). The first case of acute myocardial infarction due to a penicillin allergy was reported by Pfister and Plice in 1950. In 1991, Greek scholars Kounis et al. established a correlation between allergic reactions, inflammatory mediators, and concurrent angina pectoris, thereby proposing the concepts of “allergic angina syndrome” and “allergic myocardial infarction” (2). Therefore, many scholars started referring to the acute coronary syndrome induced by severe allergic reactions as Kounis Syndrome in subsequent reports. KS is not a rare condition and can occur at any age. However, due to its atypical clinical manifestations and scarce clinical data, clinicians’ knowledge of KS remains incomplete, resulting in frequently underdiagnosed conditions in clinical practice (3).

## Case report

**Figure 1:**
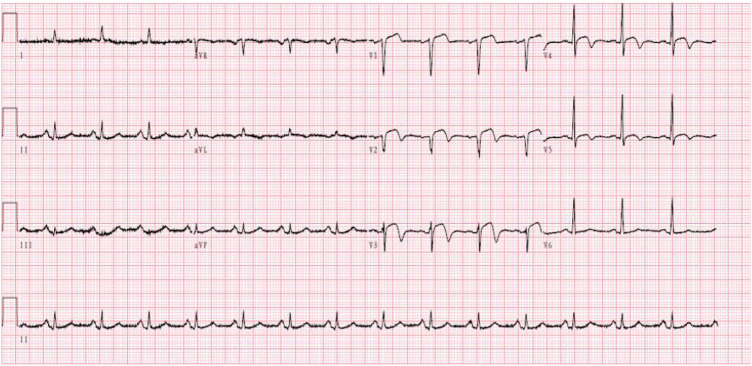
Electrocardiogram showing extensive anterior wall myocardial infarction.

**Figure 2:**
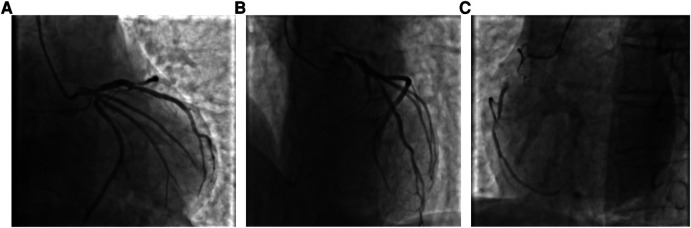
(**A**) Coronary angiogram showing limited stenosis in the proximal segment of the LAD, up to 95% or more, with antegrade TIMI grade 3; (**B**) Coronary angiogram showing no significant stenosis in the LCX, with antegrade TIMI grade 3; and (**C**) Coronary angiogram showing diffuse stenosis from the proximal to the middle and distal segments of the RCA, up to 90% or more, with antegrade TIMI grade 3.

**Figure 3:**
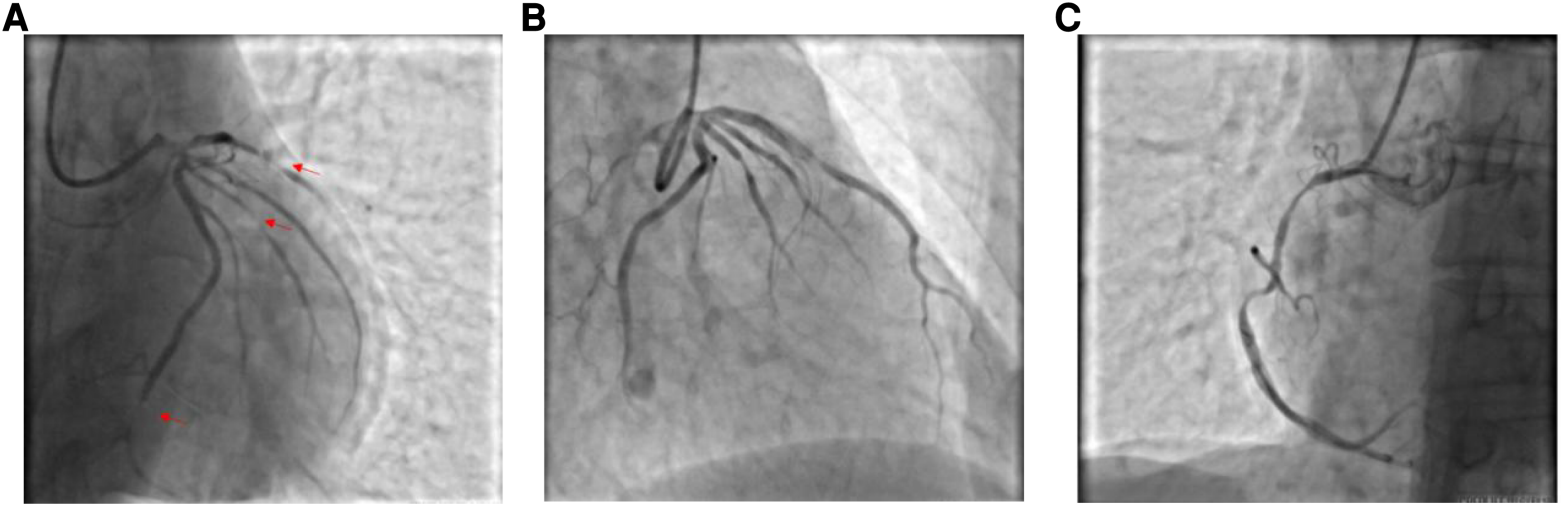
(**A**) Coronary angiography shows proximal occlusion of the LAD, stenosis of the OM and LCX, and diffuse stenosis of the RCA throughout, with occlusion of the distal segments. (**B**) Stenosis is relieved by intracoronary injection of nitroglycerin. (**C**) Stenosis of the RCA is markedly improved by administration of nitroglycerin for half a minute.

**Figure 4:**
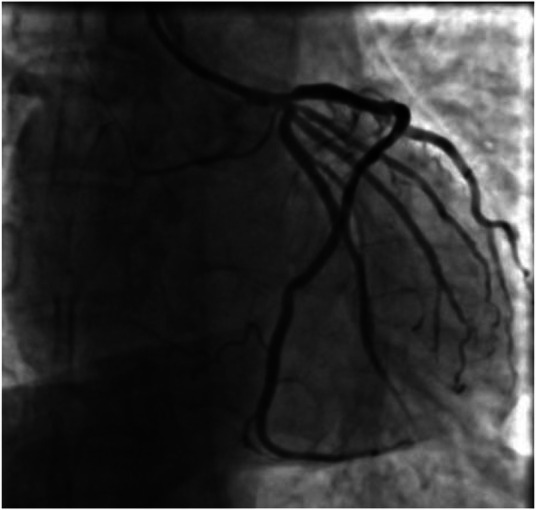
Coronary angiogram showing no residual stenosis after placement of 1 stent in the proximal segment of the LAD with anterograde flow TIMI grade 3.

A 59-year-old male was admitted to our center, complaining of persistent chest pain over three days. He reported a 40-year history of heavy smoking and sporadic alcohol consumption for 18 years. His medical, allergy, and family histories were unremarkable. On admission, myocardial injury biomarkers revealed a myoglobin level of 45.4 ng/ml (0–121 ng/ml), a CK-MB mass of 4.85 ng/ml (0–3.38 ng/ml), and an ultrasensitive troponin I level of 2.47 ng/ml (0–0.034 ng/ml). The electrocardiogram (MAC800 model, GE Company) revealed an acute, extensive anterior wall myocardial infarction (Figure 1). Further, an echocardiogram (vividE9 model, GE Company) identified a left ventricular ejection fraction amounting to 56%, coupled with segmental motion abnormalities of the ventricular wall and a decreased amplitude of the lower ventricular septal pulsation. Following the diagnosis of “coronary artery atherosclerotic heart disease, extensive anterior myocardial infarction with Killip grade I classification”, a coronary angiogram was executed on the fifth day of the patient's hospital stay. Local stenosis of 95% within the proximal Left Anterior Descending artery (LAD) was evident, with a Thrombolysis in Myocardial Infarction (TIMI) flow grade of 3 (Figure 2A). Meanwhile, the Left Circumflex artery (LCX) showed non-significant stenosis, maintaining a TIMI grade of 3 (Figure 2B). Moreover, diffuse stenosis from the proximal to middle and distal segments of the Right Coronary Artery (RCA) was noted, along with greater than 90% stenosis, preserving a TIMI grade 3 flow (Figure 2C). We proposed to perform stenting for LAD, but the patient had a sudden onset of a peripheral red rash, decreased intracoronary pressure, and dyspnea during angiography. Re-evaluation angiography revealed proximal LAD occlusion, stenosis of the Obtuse Marginal artery (OM) and LCX, and diffuse RCA stenosis with distal segment occlusion (Figure 3A). The patient's blood pressure dropped to 55/35 mmHg, while his heart rate was 108 bpm. We attributed this to an allergic reaction and promptly administered adrenaline 0.1 mg, dexamethasone 10 mg, morphine injection 3 mg immediately, and norepinephrine infused to relieve the allergic reaction and maintain blood pressure. At the same time, continuous and large amounts of intracoronary nitroglycerin was given, after which the patient's symptoms gradually subsided, stenosis improved (Figure 3B), and RCA stenosis improved significantly after half a minute of nitroglycerin administration (Figure 3C). Following a mid-segment LAD stent implantation, blood flow was restored (Figure 4). Postoperatively, his heart rate was 103 bpm, his blood pressure was 107/63 mmHg, and he was transferred to the coronary care unit for further surveillance. Postoperative examination: the patient was clear and cooperative,but a generalized red rash remained. He was diagnosed with Type II Kounis Syndrome with cardiogenic shock induced by iodophorol. His management included promethazine, vitamin C, norepinephrine and dopamine, after which the patient's blood pressure was maintained at 100/60 mmHg. On the first postoperative day, his blood pressure was 106/63 mmHg, his pulse was 75 bpm, and his level of consciousness was normal. The red rash gradually faded. Laboratory studies exhibited a cardiac troponin I level of 0.582 ng/ml (0–0.034 ng/ml) and an elevated high-sensitivity C-reactive protein level of 87.52 mg/L (0–3.5 mg/L). His IgM level was normal at 0.55 g/L (0.3–2.2 g/L), and his IgE level was less than 18.80 IU/ml (<100 IU/ml). Four days after the surgical intervention, the patient reported no discomfort, and his ultrasensitive troponin I level was down to 0.159 ng/ml (0–0.034 ng/ml). He was discharged with instructions for regular antiplatelet medication and future caution with contrast agents.

### Epidemiology

A prospective study by Ayhan Akoz and colleagues suggested that the incidence of KS in the emergency department was 19.4 cases per 100,000 individuals per year among all patients admitted to the hospital. This rate reflects the under-recognition and under-reporting of this condition rather than its rarity (4). Moreover, a large epidemiological study from the U.S. revealed that among 235,420 patients admitted due to allergic or hypersensitivity reactions, 2,616 patients (1.1%) presented with acute coronary syndrome and were subsequently diagnosed with KS (5). Although KS can manifest at any age, it predominantly affects males aged 40–70 years (74.3%). Individuals with a history of allergies, hypertension, smoking, diabetes mellitus, and hyperlipidemia are at increased risk of developing KS. Of all the reported KS cases, 6% are induced by contrast medium (6).

### Etiology and pathophysiological mechanisms

The exact pathophysiological mechanisms contributing to Kounis syndrome (KS) are unknown. Mast cells, abundant in both the heart and blood vessels, are central to the pathogenesis of KS. Their excessive production and breakdown have been implicated in the pathophysiology of coronary artery vasospasm (7). The degranulation of mast cells leads to the release of allergic inflammatory mediators such as histamine, proteases, thromboxane, prostaglandins, leukotrienes, 5-hydroxytryptophan, and various cytokines. These contribute to coronary artery constriction, plaque rupture, and platelet aggregation, all of which play an important role in KS-associated angina (8). Mast cell activation in Acute Coronary Syndromes (ACS) is considered a primary event,not a result of coronary artery spasm. There is evidence that tryptase levels are elevated in peripheral blood during spontaneous myocardial ischemia, whereas they are not elevated during drug-induced coronary artery spasm, this situation also proves it (9). Literature suggests that while any drug can potentially induce KS, common culprits include antibiotics, analgesics, anesthetics, anti-tumor drugs, herbal medicines, and contrast media (10). Notably, some studies have indicated that iodinated contrast agents applied for coronary angiography can also induce KS during secondary patient exposure (11).

### Diagnosis

The spectrum of initial symptoms in Kounis Syndrome (KS) is wide, ranging from mild indicators (e.g., flushing, nausea, vomiting, chest pain, and chest discomfort) to severe instances (e.g., hemodynamic instability and sudden death) (12). In any patient presenting with systemic allergic reactions, if these are coupled with acute myocardial ischemic symptoms reflected in clinical examinations, electrocardiograms, echocardiograms, angiographic assessments, and laboratory studies, a diagnosis of KS should be contemplated. Complementary diagnostic tests like cardiac magnetic resonance imaging, optical coherence tomography, and myocardial scintigraphy can also aid in the diagnosis. Serum trypsin-like enzymes, IgE antibodies, cardiac enzymes, Creatine Kinase (CK), CK-MB, and troponin should be tested in all cases to confirm or exclude the diagnosis of KS (13). In cases presenting with shock, the onset of an allergic reaction may be so rapid that rashes may not appear. Consequently, the absence of a rash does not exclude an allergic reaction but could be indicative of hypotensive shock (14). Now most scholars are recognized to classify KS into three types: Type I, the most prevalent, typically occurs in patients with intact coronary arteries devoid of risk indicators for coronary artery disease. This condition is triggered by allergen-induced inflammation, releasing inflammatory mediators that give rise to coronary artery spasms, occasionally accompanied by elevated myocardial enzymes and troponin levels. Type II emerges as an acute myocardial infarction induced by coronary artery spasms in patients with pre-existing coronary artery disease, frequently correlating with plaque erosion or rupture. Type III, a variant, materializes in patients with a past record of coronary artery stenting, where allergic reactions instigate acute thrombus formation within the stent (15).

### Treatment progress

The management of Kounis Syndrome (KS) must simultaneously address both acute coronary syndrome and allergic reactions. As of yet, no national or international guidelines are in place; however, an immediate stoppage of potential triggers such as medications, food, and environmental exposures can prevent further cardiac injury (16). Treatment for Type I KS often includes simple anti-allergic measures such as corticosteroids (for example, hydrocortisone) and H1 and H2 receptor antagonists, which often suffice to alleviate symptoms. For Type II KS, treatment should be with both corticosteroids and antihistamines and follow ACS treatment guidelines. In patients with type III KS, a severe myocardial infarction protocol should be followed and thrombus aspiration performed immediately, followed by histologic examination of the aspirated material and staining for eosinophils and mast cells (17). In patients who develop allergic reactions post-stent implantation, standard anti-allergic treatments can often be effective. Should these measures fail, the causative agent can be identified via patch and/or skin prick testing after implementing desensitization procedures. If an allergy to nitinol (nickel-titanium alloy), as confirmed by patch testing, is noted, stent removal may become necessary if desensitization fails (18). It's important to note that adrenaline, similar to beta-blockers, can exacerbate coronary artery spasms and deteriorate clinical conditions. Given these circumstances, glucagon, not adrenaline, should be the first-line therapy for treating allergic reactions in KS patients (10). Thus, adrenaline should only be administered in cases of severe allergic reactions, keeping in mind that adequate fluid resuscitation and oxygen therapy are crucial treatments for KS (19). The use of vasopressors and vasodilators to relieve coronary spasm is also contradictory in hypotensive patients. However, coronary angiography is a meaningful therapeutic approach in this setting. Firstly, both type II and type III KS are combined with acute thrombotic occlusion, requiring coronary intervention. Secondly, the intracoronary administration of nitroglycerin can alleviate vasospasms. However, the advantages must be weighed against the potential risk of exacerbating allergic reactions through the use of iodinated contrast agents (20). In cases of refractory KS, cardiopulmonary cerebral resuscitation proved ineffective, and the use of extracorporeal membrane oxygenation (ECMO) should be considered as early as possible for effective maintenance of coronary circulation (21).

## Discussion

For cardiologists, the presence of an acute coronary syndrome in patients with anaphylaxis is a challenging diagnostic dilemma (22). In the present case, the patient is a middle-aged man with no prior history of allergies or underlying coronary artery disease. The onset of disease is consistent with the prevailing epidemiologic pattern of previous KS. His condition developed into significant allergic reactions, abnormal volume distribution, and widespread peripheral vasodilation during angiography, leading to a diagnosis of iodophorol-induced Type II KS. After the patient developed allergy-related symptoms during coronary angiography, we found that the RCA stenosis was significantly worse than before and considered the possibility of KS. It has been shown that endothelial dysfunction can be detected in patients with mastocytosis and that endothelial function appears to be negatively affected by mast cell proliferation (23). Mast cells are predominantly found in the cardiovascular system at coronary plaque sites and may infiltrate areas of plaque erosion or rupture. The number of cardiac mast cells is up to 200 times higher in patients with coronary plaques compared to the coronary arteries of healthy individuals (9). Therefore, it can be hypothesized that patients with severe coronary atherosclerotic plaques are at higher risk of developing KS syndrome, even if there is no previous history of allergy. Patients without a history of allergy but with extensive mast cell activation due to advanced atherosclerosis may represent a specific type of Kounis syndrome. The treatment of this patient is ambivalent, and there are currently no harmonized guidelines on KS. Nitroglycerin can relieve coronary artery spasms but exacerbate hypotension, while adrenaline and noradrenaline can reverse hypotension but intensify coronary artery spasms. Taking into account our medical center's experience in treating KS and the treatments used in some previous case reports (24), we consider that coronary artery spasm is the underlying cause of KS. To prevent further myocardial ischemia exacerbations, in addition to the standard antiplatelet therapy for coronary artery disease, we initially administered nitroglycerin intracoronary to alleviate the coronary artery spasm during the operation. Subsequently, we employed vasoconstrictors, fluid resuscitation, and adequate anti-allergic treatment to maintain stable vital signs. The significant improvement in RCA stenosis after administration of antiallergic and intracoronary nitroglycerin treatment supports the diagnosis of iophorol-induced type II KS. The patient responded well to treatment, and his prognosis was favorable.

## Conclusion

In comparison to KS caused by other allergens, contrast-induced KS during coronary angiography poses more risk due to the potential for widespread coronary artery spasms. Prior studies have indicated that contrast-induced KS can lead to severe complications, with 23.1% of cases resulting in cardiac arrest and a 7.7% mortality rate (2). Yet, there is no clear consensus on directives for diagnosing and treating KS. Not all KS patients are effectively identified and managed in a timely manner. Presently, the diagnosis and treatment methods for KS are generally derived from cumulative experience outlined in case report summaries. Therefore, it is essential to increase the number of randomized clinical trials on KS, to improve clinicians' understanding of KS, and to incorporate the standard treatment of KS into the ACS guidelines.

## Data Availability

The original contributions presented in the study are included in the article/Supplementary Material, further inquiries can be directed to the corresponding author.
